# Optimizing the Sintering Conditions of (Fe,Co)_1.95_(P,Si) Compounds for Permanent Magnet Applications

**DOI:** 10.3390/ma17112476

**Published:** 2024-05-21

**Authors:** Jin Yiderigu, Hargen Yibole, Lingbo Bao, Lingling Bao, François Guillou

**Affiliations:** College of Physics and Electronic Information, Inner Mongolia Key Laboratory for Physics and Chemistry of Functional Materials, Inner Mongolia Normal University, 81 Zhaowuda Rd., Hohhot 010022, China

**Keywords:** hard magnetic material, permanent magnets, Fe compounds, coercivity, magnetic hysteresis, sintering

## Abstract

(Fe,Co)_2_(P,Si) quaternary compounds combine large uniaxial magnetocrystalline anisotropy, significant saturation magnetization and tunable Curie temperature, making them attractive for permanent magnet applications. Single crystals or conventionally prepared bulk polycrystalline (Fe,Co)_2_(P,Si) samples do not, however, show a significant coercivity. Here, after a ball-milling stage of elemental precursors, we optimize the sintering temperature and duration during the solid-state synthesis of bulk Fe_1.85_Co_0.1_P_0.8_Si_0.2_ compounds so as to obtain coercivity in bulk samples. We pay special attention to shortening the heat treatment in order to limit grain growth. Powder X-ray diffraction experiments demonstrate that a sintering of a few minutes is sufficient to form the desired Fe_2_P-type hexagonal structure with limited secondary-phase content (~5 wt.%). Coercivity is achieved in bulk Fe_1.85_Co_0.1_P_0.8_Si_0.2_ quaternary compounds by shortening the heat treatment. Surprisingly, the largest coercivities are observed in the samples presenting large amounts of secondary-phase content (>5 wt.%). In addition to the shape of the virgin magnetization curve, this may indicate a dominant wall-pining coercivity mechanism. Despite a tenfold improvement of the coercive fields for bulk samples, the achieved performances remain modest (*H*_C_ ≈ 0.6 kOe at room temperature). These results nonetheless establish a benchmark for future developments of (Fe,Co)_2_(P,Si) compounds as permanent magnets.

## 1. Introduction

Hard magnetic materials in general and permanent magnets in particular are indispensable functional magnetic materials of our modern life. Their importance is bound to further increase with the transition toward cleaner energy that has started in most major economies. The permanent magnet market is currently dominated in volume by low-performance yet cost-competitive ferrites (e.g., BaFe_12_O_19_ or SrFe_12_O_19_), followed by high-value rare-earth-based magnets (mostly Nd-Fe-B or Sm-Co alloys) exhibiting particularly high |*BH*|_max_ energy products. The environmental and economic costs of rare-earth extraction, separation and reduction, as well as the scarcity of some heavy rare-earths, are such that more and more attention is being paid to developing permanent magnets with a reduced rare-earth content [1,2,3,4,5]. While it will be challenging to produce rare-earth-free magnets with performances comparable to those of Nd-Fe-B or Sm-Co materials, one of the proposed strategies is to develop cost competitive magnets with performances somewhat intermediate between ferrites and rare-earth magnets [6,7]. These “gap” magnets should therefore be based on raw materials whose market availability is perceived as non-critical, while they should also outperform the existing ferrites. Several potential material families have been identified, a few of them attracting a dedicated interest for further developments, including Mn-based magnets such as MnAl and MnBi compounds [8,9,10,11] or Co-based alloys including Hf-Co and Zr-Co [12,13,14,15]. Among the potential hard magnets based on Fe, Fe_2_P compounds stand out for their relatively large magneto-crystalline anisotropy and significant saturation magnetization [16].

Research on permanent magnets deriving from Fe_2_P actually dates back to 1962, when (Fe,Co)_2_P powders prepared by lixiviating in Cu melt demonstrated sizable coercive fields at room temperature (*H*_C_~2 kOe) [17]. Alternative preparation methods such as plasma atomization has led to an improvement of the coercivity, reaching coercive field *H*_C_ of 3.9 kOe in ultrafine Fe_1.7_Co_0.3_P nanoparticles (~28 nm) [18]. Besides technical improvements of the preparation method, efforts have also been paid to chemical compounding. The parent binary compound Fe_2_P crystallizes in a hexagonal structure with a strong uniaxial magnetocrystalline anisotropy (*K*_1_~2.4 MJ m^−3^) and a significant saturation magnetization (~120 A m^2^ kg^−1^), but the Curie temperature (*T*_C_ = 214 K) is too low for applications [19,20,21]. Co for Fe substitutions have been found suitable to increase *T*_C_ above room temperature while preserving part of the anisotropy, so that permanent magnets could be realized in (Fe,Co)_2_P ternary compounds [17,18]. This compounding strategy can, however, not be pursued at a Co:Fe ratio higher than 15:85 due to the appearance of a competing Co_2_P-type orthorhombic structure with lower anisotropy, lower transition temperature and smaller saturation magnetization [22]. Alternatively, Si, B or As for P metalloid substitutions are also known to significantly increase *T*_C_ above room temperature, but they too result in a decreased magnetocrystalline anisotropy, the appearance of solubility limits (B) or competing crystal structures (Si and As) [23,24]. Simultaneous metal and metalloid substitutions have been theoretically proposed to overcome some of the limitations of ternary compounds and maintain a uniaxial magnetocrystalline anisotropy while increasing the Curie temperature [25]. Recent experimental studies in bulk (Fe,Co)_2_(P,Si) polycrystalline materials have indeed shown that simultaneous metal and metalloid substitutions can raise the Curie temperature (*T*_C_ up to 650 K) while maintaining a relatively large *c*-axis uniaxial magnetocrystalline anisotropy and the desired hexagonal Fe_2_P-type structure [26,27,28]. Single-crystal studies have confirmed the combination of significant room-temperature anisotropy (*K*_1_ in the range 0.9 to 1.1 MJ m^−3^), sizable saturation magnetization (corresponding to saturation polarization of 0.8–1.0 T at room temperature) and high Curie temperatures, making (Fe,Co)_2_(P,Si) quaternary alloys intrinsically promising for permanent magnet applications [29,30]. Unfortunately, neither (Fe,Co)_2_(P,Si) bulk polycrystalline materials nor single crystals present a noticeable magnetic coercivity at room temperature.

As frequently observed in permanent magnets, a coercivity mechanism combining both the intrinsic properties of the material and the actual microstructure of the samples is required to give rise to hard magnetic properties. Unfortunately, the technical methods to achieve it are unique to each materials family. In (Fe,Co)_2_P ternary compounds, a shaping into fine or ultrafine particles at the synthesis stage by Cu lixiviating or plasma atomization was found suitable to induce coercivity [17,18]. Similarly, high-energy ball milling of (Fe,Co)_2_(P,Si) bulk polycrystalline samples into submicron sized particles *after* the sintering stage was reported to induce a coercivity of *H*_C_ ≈ 1.4 kOe [29]. However, this post-sintering ball-milling approach is not ideal. It is a lengthy process that is difficult to upscale and the coercivity remains particularly low compared to the anisotropy field (*H*_a_), with a coercive field over anisotropy field ratio of, typically, *H*_C_/*H*_a_~3% [29]. One should seek alternative methods to prepare (Fe,Co)_2_(P,Si) samples while ensuring a microstructure compatible with hard properties. Here, we aim to optimize the synthesis route encompassing a ball-milling stage followed by a solid-state reaction. While this method is commonly employed for preparing Fe_2_P magnetocaloric compounds, it should be optimized to fit the specific requirements of hard magnetic materials. In particular, we seek to take advantage of the initial high-energy ball-milling stage to shorten the sintering stage. When synthesizing Fe_2_P compounds, the ball milling before sintering is typically carried out for 10 h and yields a fine, mostly amorphous, chemically homogeneous reactive mixture, requiring lower temperatures and shorter sintering for the solid-state reaction to occur than traditional methods based on mixing elemental precursors in an agate mortar. We note that this 10 h ball-milling step is not strictly speaking a mechanochemical synthesis, since the later would require one order of magnitude longer milling times (~100 h) before signatures of an Fe_2_P-type phase appear on the XRD of powders obtained from milling without sintering [31,32]. By optimizing the sintering temperature and minimizing the sintering time just after the ball-milling stage, we seek to form Fe_1.85_Co_0.1_P_0.8_Si_0.2_ compounds while preventing grain growth. Finally, we note that short time annealing (a few minutes to half an hour) or low sintering temperatures are not unusual in the preparation of hard magnetic materials with fine microstructure [33,34,35,36,37], so that this possibility is worth exploring in (Fe,Co)_2_(P,Si) compounds.

## 2. Materials and Methods

A metal deficient nominal composition Fe_1.85_Co_0.1_P_0.8_Si_0.2_ is selected for this study, so as to limit the formation of secondary phases having a metal-to-metalloid ratio of 3 to 1 [26]. A batch of Fe_1.85_Co_0.1_P_0.8_Si_0.2_ powder (50 g, Fe powder (>99.9%), Co powder (>99.9%), P powders (>98.9%) and Si lumps (>99.999%); all reactants originate from Alfa Aesar, Haverhill, MA, USA) was prepared by high-energy planetary ball milling, over 10 h, of elemental starting materials and using a sample-to-ball mass ratio of 1 to 5 (Fritsch, Idar-Oberstein, Germany, Pulverisette 5, 80 mL grinding bowls, hardened stainless steel bowls and balls). The resulting powder was then shaped by uniaxial compaction at 500 MPa into a cylinder and then sealed in a quartz ampule backfilled with 200 mbar Ar. After a hot insertion, different sintering durations (*t*_sinter._ = 15 s, 30 s, 2 min, 5 min and 10 min) and different furnace temperatures considered hereafter as the nominal sintering temperatures (*T*_sinter._ = 700 °C, 800 °C, 900 °C, 1000 °C and 1100 °C) were used and followed by a quenching in room-temperature water (no breaking of the ampule). This led to the investigation of 25 different samples originating from the same feeding powder.

**Figure 1:**
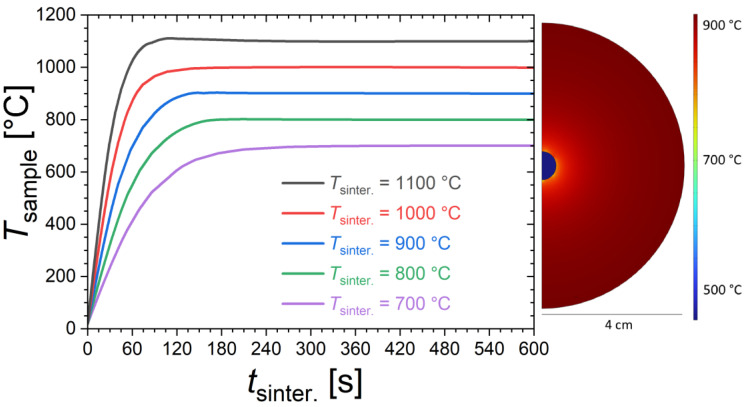
Time dependence of the sample temperature (*T*_sample_) for different furnace temperatures, estimated from finite element simulations. On the right, illustration of the temperature distribution calculated from finite element simulations at *t*_sinter._ = 30 s for a furnace temperature of *T*_sinter._ = 900 °C.

In view of the relatively short sintering, the actual temperature reached in the sample can be different from the nominal sintering temperature (*T*_sinter._) from the furnace. Finite element simulations were performed to provide a rough estimate of the sample temperatures. Figure 1 shows the time dependence of the sample temperature for different furnace temperatures calculated using the COMSOL Multiphysics software (version 5.6) with heat transfer package (heat transfer in solids and fluids with surface-to-surface radiation), considering the actual sample and furnace dimensions in a transient 2D model including conduction, natural convection and radiative heat transfers. We acknowledge that these simulations are based on particularly crude assumptions, so that their interpretation should be limited to discussing orders of magnitude and tendencies. In particular, the solid-state reaction occurring in the sample is neglected; for instance, sample shape (shrinkage is neglected), density, heat capacity, thermal conductivity and surface emissivity are taken as constant as a function of the temperature. The simulations suggest that sintering durations of 2 to 3 min are typically required to reach the targeted furnace temperature. Temperature homogenization is actually the fastest at the highest furnace temperatures due to the dominant role played by radiative heat transfers. The thermal gradient within the sample is found to be negligible compared to the difference between sample and furnace temperatures. This latter point was experimentally verified since no significant differences in structural or magnetic properties were observed between pieces selected from the core or from the surface of the pellet. We also note that the finite element simulations are reasonably in line with qualitative observations made during the quenching stage. For instance, for the sintering at 1100 °C, the sample sintered for only 15 s shows only a very faint red glow during quenching, which is typical of temperatures lower than 500 °C; meanwhile, the sample sintered for 60 s shows a bright orange glow at the quenching stage, which is typical of temperatures higher than 900 °C.

Powder X-ray diffraction (XRD) experiments were carried out using an Empyrean PANalytical diffractometer (Malvern Panalytical, Malvern, UK) using Cu Kα radiation, a PIXcel detector and a typical collection time of 1 h per sample. The Rietveld method, as implemented in the FullProf software, (version April 2019) is used for structural analysis [38]. Specific magnetization versus applied magnetic field measurements were performed in a Versalab system (Quantum Design China, Beijing, China) equipped with a vibrating sample magnetometer option. The measurements were carried out while ramping the magnetic field at 20 Oe s^−1^ and using a 1 s acquisition time. In the field range relevant for the determination of the coercive field, the field increments are larger than the uncertainty due to the field uniformity (±0.1%) or the power supply stability (≈0.5 Oe). The field increment is therefore the main parameter limiting the accuracy of the determination of *H*_C_ to ±20 Oe. The measurements were carried out on bulk polycrystalline pieces having a cubic shape (no demagnetization correction) and a typical mass of ~20 mg.

## 3. Results and Discussion

**Figure 2:**
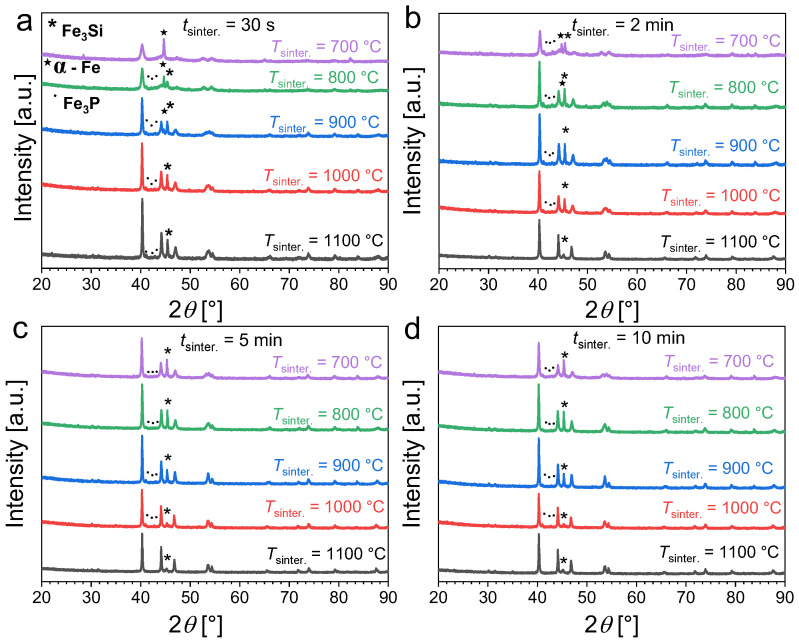
Powder X-ray diffraction patterns measured at room temperature for Fe_1.85_Co_0.1_P_0.8_Si_0.2_ powders sintered at different temperatures and for various sintering durations (30 s panel (**a**), 2 min panel (**b**), 5 min panel (**c**) and 10 min panel (**d**)).

Figure 2 presents the powder X-ray diffraction patterns of Fe_1.85_Co_0.1_P_0.8_Si_0.2_ powders as a function of the sintering time (*t*_sinter._) and temperature (*T*_sinter._). For short sintering durations (30 s or less), a moderate sintering temperature of 700 °C does not allow for the solid-state reaction to occur as only a minor amount of product is formed while a significant fraction of unreacted α-Fe is still observed. This primarily originates from the large difference between the nominal furnace temperature and the actual sample temperature for the shortest sintering; see Figure 1. Increasing the temperature favors the formation of the desired hexagonal phase with an Fe_2_P crystal structure. After the ball-milling stage, sintering for 30 s at 1100 °C is sufficient to obtain an Fe_1.85_Co_0.1_P_0.8_Si_0.2_ sample mostly composed of the desired Fe_2_P phase, but non-negligible amounts of secondary Fe_3_P (~6 wt.%) and Fe_3_Si (~9 wt.%) phases remain detectable. Increasing the sintering duration also favors the formation of the Fe_2_P-type phase. For sintering at 700 °C, increasing the duration progressively increases the Fe_2_P-type content; but even after 10 min sintering non-negligible amounts of secondary phase are still observed. High temperatures (sintering at or above 1000 °C) are required to reduce the secondary-phase content. Sintering at 1100 °C for 5 to 10 min allows the preparation of Fe_1.85_Co_0.1_P_0.8_Si_0.2_ samples with a reasonable purity (with approximately 5 wt.% of Fe_3_Si secondary phase).

**Figure 3:**
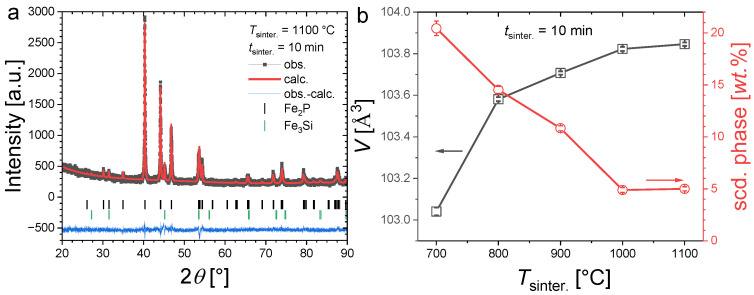
Panel (**a**), Illustration of a refined XRD pattern measured at room temperature for Fe_1.85_Co_0.10_P_0.8_Si_0.2_ sintered at 1100 °C for 10 min. Panel (**b**), Unit cell volume and secondary-phase content of Fe_1.85_Co_0.10_P_0.8_Si_0.2_ samples sintered during *t*_sinter._ = 10 min at different temperatures.

Figure 3a illustrates the refinement of a typical powder XRD pattern for an Fe_1.85_Co_0.1_P_0.8_Si_0.2_ sample containing a secondary phase. Figure 3b shows the evolution of the unit cell volume and secondary-phase content for 10 min sintering at different temperatures. After sintering at 700 °C for 10 min, the secondary-phase content is particularly large (~21 wt.%). Increasing the sintering temperature allows one to reduce the Fe_3_Si secondary-phase content, which also results in a cell volume increase for the main phase. This evolution is pronounced from 700 °C to 1000 °C and becomes much less marked from 1000 °C to 1100 °C. This result is in line with former studies on the synthesis of (Fe,Co)_2_(P,Si), indicating that a relatively high sintering temperature (1100 °C, corresponding to approximately 85% of the incongruent melting temperature) is required to avoid the formation of competing Fe_3_P or Fe_3_Si phases [26]. However, in contrast to former reports employing the sintering of several hours or days, here we show that a few minutes sintering at high temperatures are sufficient to form samples with the desired hexagonal Fe_2_P-type structure with limited secondary phases.

**Figure 4:**
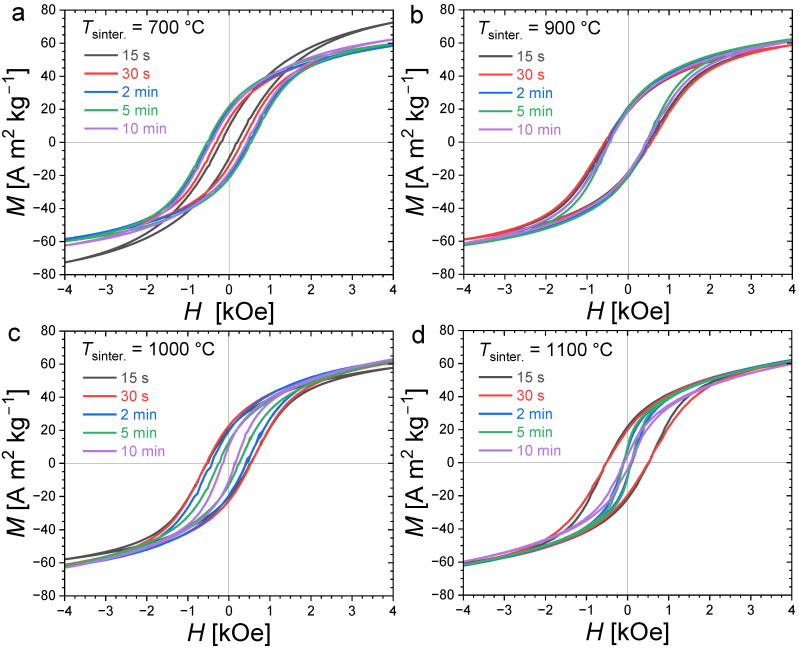
Room-temperature (*T* = 300 K) magnetization hysteresis curves for Fe_1.85_Co_0.10_P_0.8_Si_0.2_ bulk polycrystalline samples sintered at different temperatures (700 °C, 900 °C, 1000 °C and 1100 °C in panel (**a**), (**b**), (**c**) and (**d**), respectively) and durations (from 15 s to 10 min).

Figure 4 presents magnetization hysteresis cycles measured at *T* = 300 K for polycrystalline bulk Fe_1.85_Co_0.10_P_0.8_Si_0.2_ samples sintered at different temperatures and for various durations (randomly oriented). All samples present an opening between their magnetization/demagnetization curves typical of hard or semi-hard magnetic materials. Large differences in coercivity can, however, be observed; in particular, the evolution of *H*_C_ as a function of the sintering duration shows a stark contrast for different annealing temperatures. When the sintering is carried out at 700 °C, the smallest coercivity is observed for the shortest sintering duration. Increasing the sintering time leads to an increased coercivity until of approximately 2 min, then *H*_C_ stabilizes at approximately 0.54 kOe. At 900 °C, the hysteresis cycles nearly overlap. The sintering duration has only a limited influence on the coercive fields at this sintering temperature, and it corresponds to the observation of the largest coercivities (0.58 kOe). In contrast, when sintering at higher temperatures, such as 1000 °C or 1100 °C, the coercive field tends to decrease, with an increase in the sintering time.

**Figure 5:**
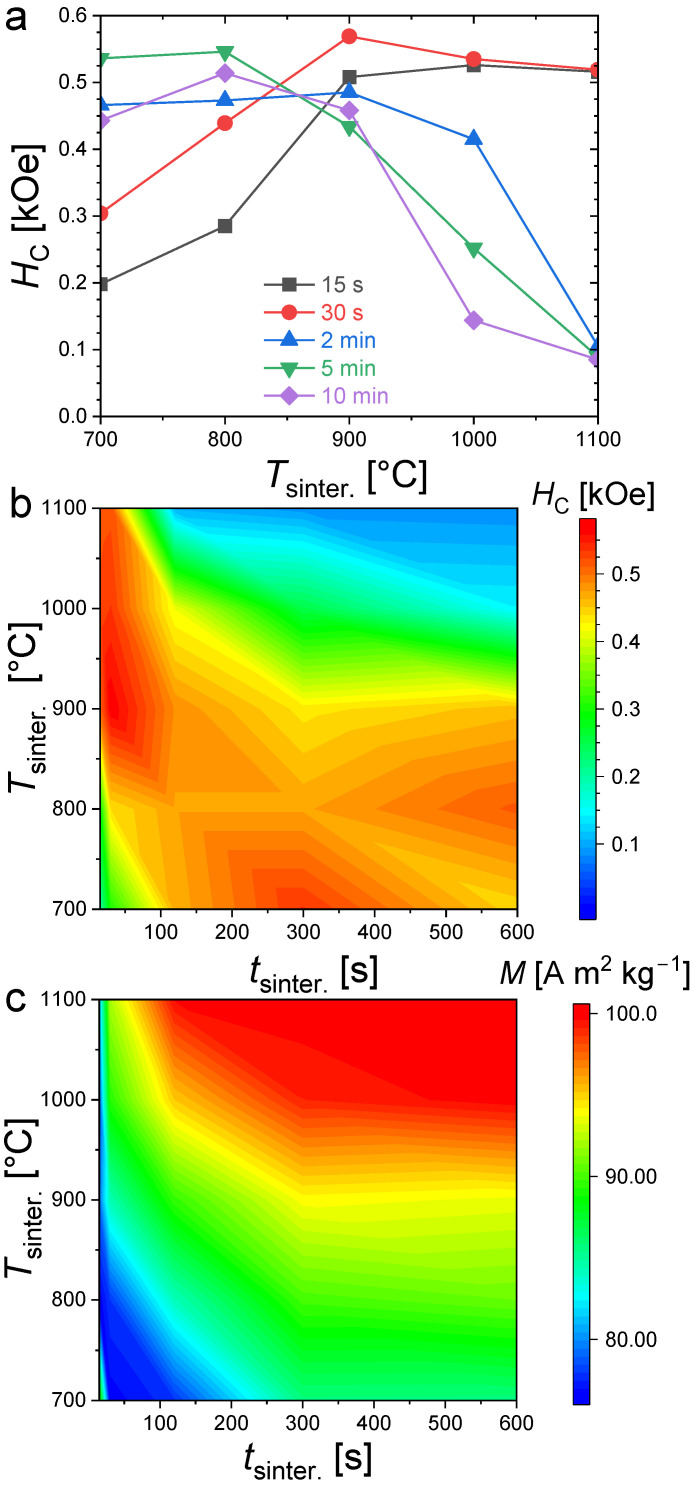
Coercive fields (**a**,**b**) at *T* = 300 K and magnetization at *T* = 300 K and *H* = 30 kOe (**c**), for Fe_1.85_Co_0.10_P_0.8_Si_0.2_ bulk samples sintered at different temperature and for various time.

Figure 5 summarizes the evolution of the coercivity and saturation magnetization (magnetization taken at *T* = 300 K and *H* = 30 kOe). The large differences between the coercive field and saturation magnetization well illustrate the specificities of the coercivity optimization. First, let us set aside the case of the shortest 15 s and 30 s sintering times at the lowest sintering temperature of 700 °C, which shows an out of trend saturation magnetization. This abnormally high magnetization compared to that of the surrounding samples is likely originating from the high α-Fe content detected in powder XRD, since the latter has a room-temperature saturation magnetization (~220 A m^2^ kg^−1^) significantly larger than that of (Fe_,_Co)_2_(P,Si) compounds. Increasing the sintering time at 700 °C favors the formation of the Fe_2_P-type phase, which triggers a reduction in saturation magnetization and an increase in coercivity. More generally, increasing the sintering time or increasing the sintering temperature allows one to reach higher magnetization since it results in larger phase fractions and better crystallized of Fe_2_P-type products (with the exception of α-Fe, the saturation magnetizations of the other secondary phases are lower than that of Fe_2_P, ~1.70 μ_B_/f.u. for Fe_3_P, that is to say, ~48 A m^2^ kg^−1^ [39] or ~137 A m^2^ kg^−1^ for Fe_3_Si at 5 K, but significantly lower at room temperature [40,41]).

The coercivities show a very different distribution than the saturation magnetization as a function of the sintering conditions (see Figure 5). High coercivities tend to form a strip starting for the shortest sintering times at the highest temperatures and broadening toward longer sintering at lower temperatures. It is particularly interesting to point out that the largest coercivities are not observed in the purest samples synthesized at high temperatures (*T*_sinter._ ≥ 1000 °C) for a long time (*t*_sinter._ ≥ 5min). The largest coercivities are rather observed in the (Fe,Co)_1.95_(P,Si) samples presenting significant amounts of secondary phases. For instance, all the samples synthesized at 900 °C (from 15 s to 10 min sintering) present a large contamination of cubic Fe_3_Si secondary phase (>10 wt.%) plus traces of Fe_3_P for a few samples. At a given sintering duration, the Fe_2_P-type phase content is less in samples sintered at 900 °C than that of the sample sintered at 1100 °C, and yet they show a larger coercivity.

**Figure 6:**
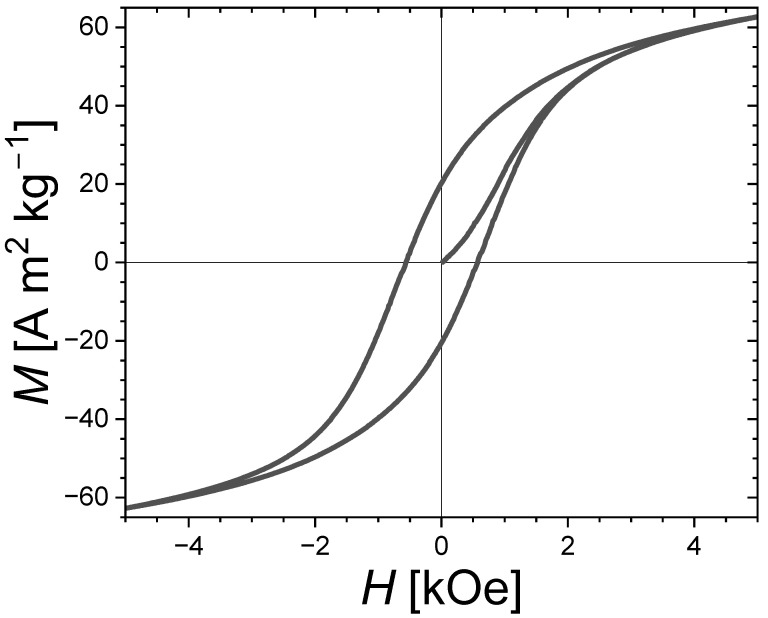
Magnetic hysteresis cycle with the first magnetization curve at *T* = 300 K for the Fe_1.85_Co_0.10_P_0.8_Si_0.2_ bulk sample sintered for *t*_sinter._ = 30 s at *T*_sinter._ = 900 °C. A “non-magnetized” state was ensured by performing a demagnetization in evanescent-applied magnetic fields prior to the measurements.

The secondary phases of Fe_3_P and Fe_3_Si have only a modest magnetocrystalline anisotropy compared to that of the main Fe_2_P-type phase and are therefore not anticipated to be directly responsible for the hard magnetic properties. Nevertheless, the presence of soft secondary phases appears to favor the development of coercivity. It is even tempting to establish a similarity with the wall-pining coercivity mechanism occurring in some nanocomposite magnets involving intergranular phases. The virgin magnetization curve presented in Figure 6 for an Fe_1.85_Co_0.10_P_0.8_Si_0.2_ bulk polycrystalline sample presents an *S*-shape with a critical field close to that of the coercive field, which is rather typical of a domain wall-pinning mechanism. While further insights into the coercivity mechanism would be needed to ascertain it, we may nonetheless point out that the structural and magnetic inhomogeneities due to secondary phases impeding the domain wall motion could well be responsible for observing the largest coercivities in the samples with large amounts of Fe_3_P and/or Fe_3_Si secondary phases. In addition, former studies in (Fe,Co)_2_P ternary compounds observed a coercivity optimum for the Co content corresponding to the Fe_2_P-type/Co_2_P-type structural boundary [17,18], and in sub-micron-sized (Fe,Co)_1.95_(P,Si) particles the largest coercivity was observed in a sample presenting a two-phase mixture of Fe_2_P-type and orthorhombic BCO-type structures [29]. These observations could also be in line with a pinning-type coercivity favored by structural disorder and large secondary-phase contents.

From a quantitative point of view, the present samples prepared using short sintering show tenfold larger coercivities (*H*_C_ ≈ 0.58 kOe) than that found in bulk polycrystalline samples sintered for 24 h (*H*_C_ typically less than 50 Oe) or single crystals (*H*_C_ typically less than 20 Oe) [26,29,30]. However, the coercivities are twice as small as that observed in sub-micron-sized (Fe,Co)_2_(P,Si) particles obtained by ball milling (ball-milling stage *after* a solid-state synthesis, *H*_C_ up to approximately 1.4 kOe at room temperature [29]). The present coercivities also remain considerably lower than the anisotropy field (*H*_A_ of approximately 28 kOe for the present composition [29]), indicating that short sintering after ball milling, while convenient to implement in practice, is not an ideal method to turn the high intrinsic potential of (Fe,Co)_2_(P,Si) quaternary compounds into permanent magnet applications. Pragmatically, the present coercivities are smaller than those of other rare-earth-free materials or of ferrite magnets. The maximal energy product, |*BH*|_max_, is approximately 2.0 kJ m^−3^ for the present isotropic bulk samples, which is of the same order of magnitude yet less than that of isotropic ferrites (5 kJ m^−3^ typical). This study nonetheless confirms that the development of alternative synthesis methods and specific microstructures remains a priority in order to turn the promising intrinsic properties of the (Fe,Co)_2_(P,Si) material family into actual permanent magnets. Observing a significant coercivity in short-sintered samples is an additional indication that limiting grain growth is beneficial to the coercivity. Further attempts should therefore be paid to synthesizing (Fe,Co)_2_(P,Si) compounds while preserving a fine microstructure.

## 4. Conclusions

The structure and magnetic properties of bulk Fe_1.85_Co_0.10_P_0.8_Si_0.2_ polycrystalline samples prepared by ball milling followed by a short sintering are investigated. It is found that a few minutes of sintering at high temperatures is sufficient to form the desired Fe_2_P-type structure with a reasonable purity (95 wt.% of Fe_2_P-type phase after 10 min sintering at 1000 °C or 1100 °C). Surprisingly, the largest coercivities are not observed in the purest, well crystallized samples, but rather in those presenting significant amounts of secondary phases. This observation, as well as the shape of the virgin magnetization curve, may suggest a dominant pining mechanism. The optimization of the sintering leads to an improvement by one order of magnitude of the coercivity in isotropic bulk Fe_2_P-based materials. However, the achieved coercivities remain small compared to the anisotropy field. (Fe,Co)_2_(P,Si) compounds deserve further investigation. First, local magnetization measurements would be needed to better describe the role of the secondary phases in promoting coercivity and resolving the nucleation mechanism. Then, alternative synthesis methods to create fine microstructures should be sought for, for instance, by using fast cooling processes such as the melt-spinning method or by using other sintering techniques with a better control of the temperature profile, such as microwave sintering or spark plasma sintering methods.

## Data Availability

Dataset available on request from the authors.
